# Responsiveness of dentate neurons generated throughout adult life is associated with resilience to cognitive aging

**DOI:** 10.1111/acel.13161

**Published:** 2020-06-29

**Authors:** Marie‐Françoise Montaron, Vanessa Charrier, Nicolas Blin, Pierre Garcia, Djoher Nora Abrous

**Affiliations:** ^1^ INSERM UMR 1215, Magendie Neurocenter Neurogenesis and Pathophysiology Group Bordeaux France; ^2^ Université de Bordeaux Bordeaux France

**Keywords:** adult neurogenesis, aging cell, hippocampus, resilience, spatial memory, successful aging

## Abstract

During aging, some individuals are resilient to the decline of cognitive functions whereas others are vulnerable. These inter‐individual differences in memory abilities have been associated with differences in the rate of hippocampal neurogenesis measured in elderlies. Whether the maintenance of the functionality of neurons generated throughout adult life is linked to resilience to cognitive aging remains completely unexplored. Using the immediate early gene Zif268, we analyzed the activation of dentate granule neurons born in adult (3‐month‐old), middle‐aged (12‐month‐old), or senescent (18‐month‐old) rats (*n* = 96) in response to learning when animals reached 21 months of age. The activation of neurons born during the developmental period was also examined. We show that adult‐born neurons can survive up to 19 months and that neurons generated 4, 10, or 19 months *before* learning, but not developmentally born neurons, are activated in senescent rats with good learning abilities. In contrast, aged rats with bad learning abilities do not exhibit activity‐dependent regulation of newborn cells, whatever their birthdate. In conclusion, we propose that resilience to cognitive aging is associated with responsiveness of neurons born during adult life. These data add to our current knowledge by showing that the aging of memory abilities stems not only from the number but also from the responsiveness of adult‐born neurons.

## Graphical abstract

By imaging learning‐induced activation of dentate granule neurons born in adult, middle‐aged and old rats using a marker of neuronal activity Zif268, it was found that responsiveness of new neurons was preserved in old rats resilient to cognitive aging and not in vulnerable ones.

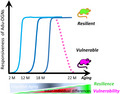

## 1 INTRODUCTION

Brain and cognition change with age, and although patterns of decline are evident at the population level, the rates of change differ among individuals as well as across brain regions and cognitive domains (Gray & Barnes, 2015; Nyberg, Lovden, Riklund, Lindenberger, & Backman, 2012). Indeed, some old individuals exhibit cognitive abilities similar to those of younger ones (optimal/successful aging) whereas others show a clear and substantial cognitive decline without signs of pathologies (suboptimal/accelerated aging). Episodic memory is particularly sensitive to aging, and investigations conducted so far both in humans and in animal models have revealed that the preservation of episodic memory abilities is correlated with the structural and functional integrity of the hippocampal formation (Gonzalez‐Escamilla, Muthuraman, Chirumamilla, Vogt, & Groppa, 2018). Several models and theories (maintenance, reserve, compensation) emerged in an effort to account for variability in cognitive outcome across old subjects, and high level of neural plasticity has been proposed for brain reserve and resilience to cognitive aging (Nyberg et al., 2012).

The ability of the adult brain, and in particular the dentate gyrus (DG) of the hippocampus, to create new neurons is a peculiar form of plasticity to protect the aging brain. Briefly, new dentate granule neurons (DGNs) generated throughout the entire life of an individual (Aimone et al., 2014), humans included (Moreno‐Jiménez et al., 2019), are integrated into functional circuits and play a crucial role in complex forms of learning and memory (Clelland et al., 2009; Dupret et al., 2008; Tronel et al., 2012). In particular, both the addition and the elimination of new neurons *before, during,* or *after* learning were found to be important for learning and forgetting in young adult rodents (Akers et al., 2014; Dupret et al., 2007).

During aging, the rate of cell proliferation (and thus neurogenesis) decreases (Drapeau & Abrous, 2008), a process associated with the progressive loss or phenotypic and functional changes of neural stem cells (NSCs), (Martin‐Suarez, Valero, Muro‐Garcia, & Encinas, 2019; Schouten et al., 2019), or their niche (Diaz‐Moreno et al., 2018). Inter‐individual differences in the rate of adult neurogenesis (ANg) have been linked to variability in spatial memory abilities of senescent animals: Preserved memory functions are associated with the maintenance of a relatively high neurogenesis level measured *after* learning, whereas memory deficits are linked to exhaustion of neurogenesis (Drapeau et al., 2003). Moreover, we have found that corticosterone dampening from middle age on has a beneficial effect on both the rate of neurogenesis and spatial memory measured once animals have reached senescence (Montaron et al., 2006). Altogether this dataset raises the fascinating hypothesis that neurons generated throughout adult life could constitute a mechanism that promotes resilience to cognitive aging.

To tackle this question, we examined whether the activation of DGNs generated throughout adult life was related to the maintenance of memory function by imaging them when animals reached senescence. DGNs born in adult (3‐month‐old), middle‐aged (12‐month‐old), or senescent (18‐month‐old) rats were labeled with analogs of thymidine, and their activation in response to spatial learning was measured using Zif268, an immediate early gene (IEG) (Tronel, Lemaire, Charrier, Montaron, & Abrous, 2015), when animals have reached senescence. The activation of DGNs born during development was also examined.

## 2 RESULTS

### 2.1 Fate of dentate granule neurons born in old rats

**Figure 1:**
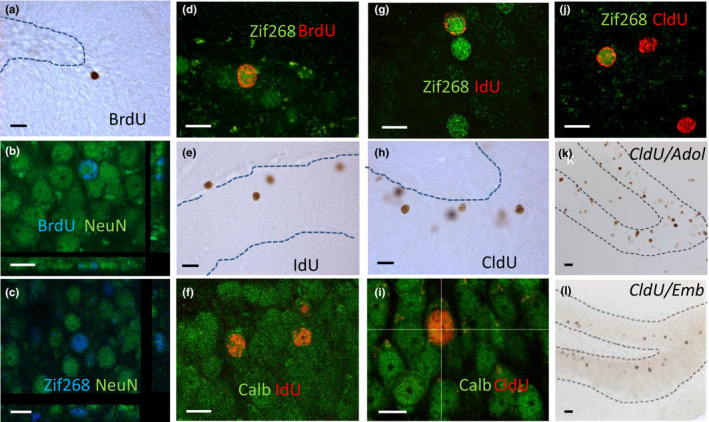
Granule neurons in the DG of aged rats. (a) Illustration of 4‐month‐old BrdU‐IR neurons in an animal with preserved memory. (b) Confocal photomicrographs of 4‐month‐old BrdU‐IR cells (blue) expressing NeuN (green). Confocal photomicrographs of (c) neurons (NeuN, green) expressing Zif268 (blue) and of (d) 4‐month‐old BrdU‐IR cells (red) expressing Zif268 (green). (e) Illustration of 10‐month‐old IdU‐IR neurons. Confocal photomicrographs of IdU‐IR cells (red) expressing (f) calbindin (green) or (g) Zif268 (green). Illustration of 19‐month‐old CldU‐IR neurons. (h) Confocal photomicrographs of CldU‐IR cells (red) expressing (i) calbindin (green) or (j) Zif268 (green). (k) Illustration of CldU‐IR neurons born in adolescent rats (PN28). (l) Illustration of CldU‐IR neurons born in embryons (ED18.5). Bar scale for DAB = 20 µm. Bar scale for confocal illustration = 10 µm

In a first step, we sought to determine whether new neurons born during *senescence* are recruited by spatial learning. To do so, 18‐month‐old rats were injected with BrdU according to a previously described protocol (Table 1) and were trained 4 months later in the water maze using a reference memory protocol (Drapeau et al., 2003). Animals were trained for 11 days (Figure S1a,b) until the aged‐unimpaired rats (AU) learned the task (day effect on Latency: *F*_11,66_ = 2.35, *p* = .016; day effect on Distance: *F*_11,66_ = 2.76, *p* = .005) and reached asymptotic levels of performances (with no statistical significant differences over the last 3 days). In contrast, the aged‐impaired (AI) rats did not learn the task although they were searching and finding the platform most of the time (2 or 3 trials out of 4) (day effect on Latency: *F*_11,66_ = 1.25, *p* = 1.25; day effect on Distance: *F*_11,66_ = 0.96, *p* = .48). Ninety minutes after the last trial, animals (and their age‐matched control group) were sacrificed for immunohistochemistry. At the time of sacrifice, BrdU‐IR cells were 4 months old and their majority was located within the granule cell layer (GCL) (Figure 1a).

**Table 1:** Summary of the procedures

Batch	Experiment	XdU	Rats’ age at XdU injections	Rats’ age at time of sacrifice	Neurons’ age at time of sacrifice	Group size
1	Recruitment of 4‐month‐old Adu‐DGNs	BrdU	18 months	22 months	4 months	C = 5 AI = 7 AU = 7
2	Recruitment of 10‐month‐old Adu‐DGNs	IdU	12 months	22 months	10 months	C = 10 AI = 11 AU = 11
	Recruitment of 19‐month‐old Adu‐DGNs	CldU	3 months	22 months	19 months	C = 10 AI = 11 AU = 11
3	Recruitment of DGNs born in adolescent rats	CldU	PN28	22 months	21 months	AI = 5 AU = 5
4	Recruitment of DGNs born in adolescent rats	CldU	PN28	14 months	15 months	AI = 5 AU = 5
5	Recruitment of DGNs born in embryos	CldU	Ed18.5	15 months	15 months	AI = 6 AU = 7

**Figure 2:**
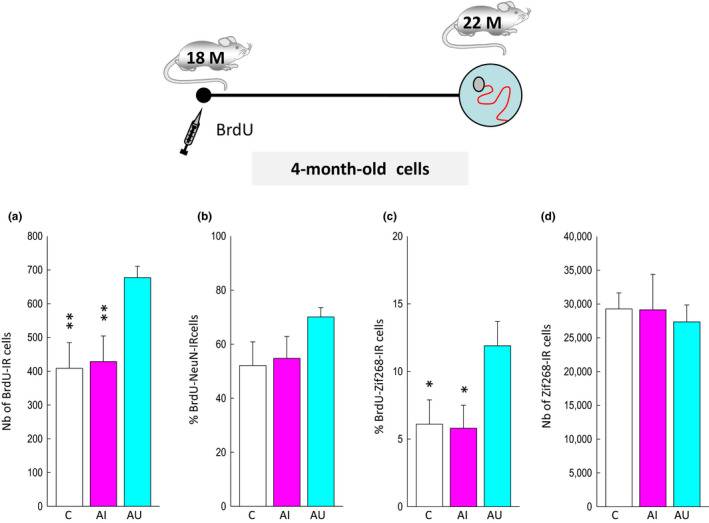
Neurons produced during old age are activated by spatial learning. Top: Experimental design. (a) The number of BrdU‐IR cells is higher in the aged rats that learned the task (AU) compared to those with spatial memory deficits (AI) or to control animals (c). (b) The percentage of cells differentiating into neurons (BrdU‐IR cells expressing NeuN) is similar in the three groups. (c) The expression of Zif268 in BrdU‐IR cells generated in senescent DG is increased in AU compared to AI rats and C rats. (d) The number of neurons expressing Zif268 is similar in the three groups. *p < .05, **p < .01 compared to AU

These cells were more numerous in the GCL of animals with good learning abilities (AU) compared to animals with memory deficits (AI) (Figure 2a, *F*_2,16_ = 7.64, *p* = .05 with C = AI<AU at *p* < .01). This finding is consistent with our previous study showing that the number of neurons generated 1 month *after* learning is higher in AU compared to AI (Drapeau et al., 2003) senescent rats. More than fifty percent of BrdU‐IR cells in the GCL expressed NeuN (Figure 1b), and neuronal differentiation was not different among groups (Figure 2b, *F*_2,16_ = 2.07, *p* = .15).

To determine whether newborn neurons are recruited by learning, we used Zif268 since this IEG is still expressed in the old DG (Gheidi, Azzopardi, Adams, & Marrone, 2013). Given that a substantial fraction of cells generated during senescence did not express NeuN, we verified in trained animals that Zif268‐expressing cells were expressing NeuN (Figure 1c). We found that the vast majority of activated cells (Zif268) were neurons (NeuN) and that this ratio was similar in good and bad learners (AI: 96.4 ± 0.5; AU: 96 ± 1.3, *p* > .05). Then, we examined the activation of adult‐born cells, meant to be neurons, in response to learning (Figure 1d). We found that the percentage of BrdU‐IR cells expressing Zif268‐IR in aged animals with good learning abilities was greater than that of aged animals with memory deficits and that of the untrained control group (Figure 2c, *F*_2,16_ = 3.70, *p* = .05 with C = AI<AU at *p* < .05). In contrast, the total number of Zif268‐IR nuclei did not differ between groups (Figure 2d, *F*_2,16_ = 0.25, *p* = .78). These results show that neuronal cells in the senescent DG are recruited by spatial learning and not by nonspecific effects of training (swimming, stress) as revealed by the lowest level of recruitment of 4‐month‐old cells in aged‐impaired and control animals.

### 2.2 Fate of dentate granule neurons born in middle‐aged and young rats

**Figure 3:**
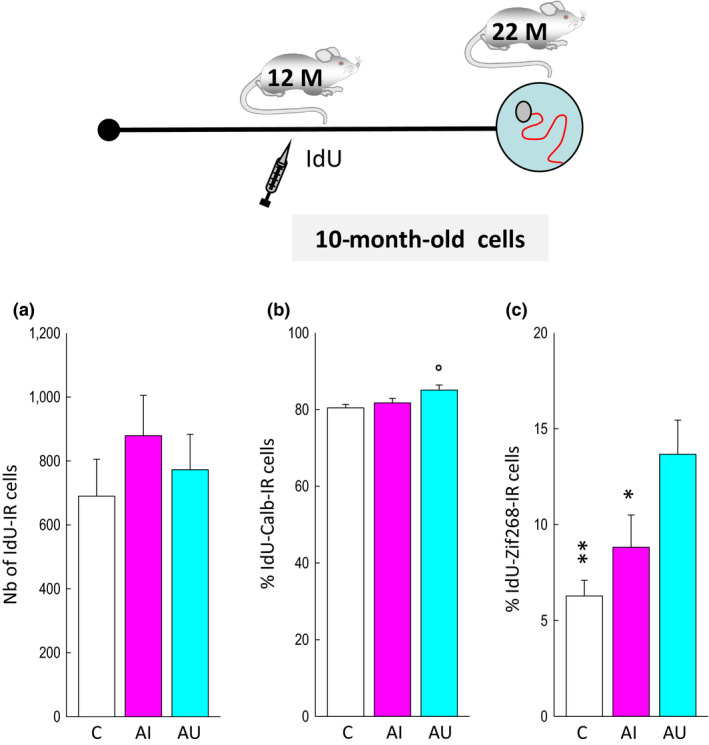
Neurons produced during middle age are activated by spatial learning in aged good learners. Top: Experimental design. (a) The number of IdU‐IR cells generated at mid‐age is independent of the memory abilities measured when rats reached senescence. (b) The percentage of cells differentiating into neurons (IdU‐IR cells expressing calbindin) is slightly increased in AI (compared to C and AU). (c) The expression of Zif268 in IdU‐IR cells is increased in AU rats compared to AI rats and C rats. *p < .05, ** < .01 compared to AU. °*p* < .05 compared to c

Then, we asked whether neurons born earlier, that is, in middle age or young adulthood, are also recruited by learning during aging. For this purpose, animals were injected with CldU when 3 months old and with IdU when middle aged (12 months old; Table 1). Animals were trained 10 months later for 11 days until the AU learned the task (day effect on Latency: *F*_10,100_ = 22.08, *p* < .001; day effect on Distance: *F*_10,100_ = 18.77, *p* < .001) and reached 3 days of stable performances (Figure S1c,d). In this batch, the AI showed a dramatic improvement of their performances on the last training day (day effect on Latency: *F*_10,100_ = 6.67, *p* < .001; day effect on Distance: *F*_10,100_ = 22.08, *p* < .001). Trained animals (and their age‐matched control group) were sacrificed 90 min after the last trial. At the time of sacrifice, IdU cells were 10 months old (Figure 1d). Their number was not influenced by training or by the cognitive status of the animals (Figure 3a, *F*_2,29_ = 0.87, *p* = .43). More than eighty percent of IdU cells expressed the neuronal marker calbindin (Figures 1f and 3b, *F*_2,28_ = 4.21, *p* = .02 with C = AI<AU at *p* = .02). The percentage of neurons born during middle age and expressing Zif268 was greater in the AU group than that measured in AI and C groups (Figures 1g and 3c, *F*_2,29_ = 4.87, *p* = .02 with C = AI<AU at *p* < .01 and *p* < .05, respectively).

**Figure 4:**
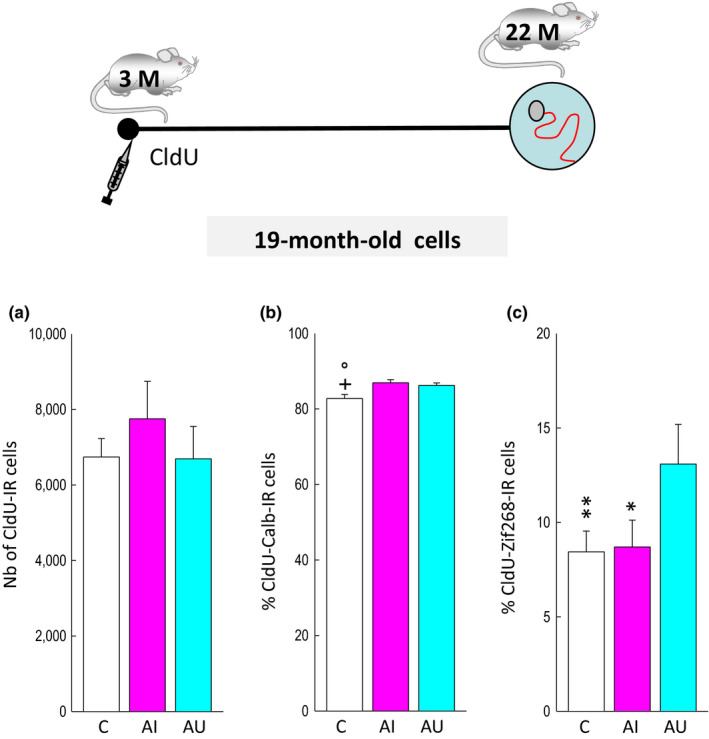
Neurons produced during young adulthood are activated by spatial learning in good leaners. Top: Experimental design. (a) The numbers of CldU‐IR cells generated when animals are young adult are independent of the memory abilities measured when rats reached senescence. (b) The percentage of CldU‐IR cells expressing calbindin is increased by training. (c) The expression of Zif268 in CldU‐IR cells generated in young adult DG is increased in AU compared to AI rats and C rats. °*p* < .05 compared to AU, +*p* < .05 compared to AI. *p < .05, **p < .01 compared to AU

Nineteen‐month‐old CldU‐IR cells were examined in the same animals (Figure 1h). Their number was not influenced by training or the cognitive status of the animal (Figure 4a, *F*_2,29_ = 0.52, *p* = .6). Their phenotypic analysis revealed that exposure to the water maze slightly increased neuronal differentiation (CldU‐calbindin co‐expressing cells; Figures 1j and 4b,c: 82.8 ± 0.1% AI: 86.9 ± 0.8%; AU: 86.2 ± 0.7%; *F*_2,29_ = 6.54, *p* < .01 with C < AI = AU at *p* = .01). Again, we found that the percentage of CldU‐IR cells expressing Zif268 was greater in the AU group than that measured in AI and C groups (Figures 1j and 4c, *F*_2,29_ = 6.96, *p* = .004 with C = AI<AU at *p* < .01 and *p* < .05, respectively). The total number of cells expressing Zif268‐IR (C: 29,270.02 ± 2,360.54: AI: 26,068.94 ± 2,366.78; AU: 28,739.22 ± 3,095.74, *F*_2,29_ = 0.42, *p* = .65) did not differ between groups.

### 2.3 Fate of dentate granule neurons born during development

Finally, we explored the role of dentate granule born during development of the DG by tagging neurons born in adolescent rats (PN28, Figure 1k) and neurons born in embryos (E18.5, Figure 1l) with CldU. Animals were sacrificed when 22 or 15 months old, and for both groups, the number of CldU‐IR cells and the percentage of CldU‐IR cells expressing Zif268 were similar in AU and AI rats (Table S1).

## 3 DISCUSSION

To determine whether neurons generated during adult life participate in learning abilities in old age, the expression of the IEG Zif268 was assessed in new neurons. We found that cells generated during young adulthood, middle age, and senescence survive for a long period of time and are functionally integrated into the dentate network. When taking into account individual differences in memory abilities, we highlight that although the number of new cells generated in 12‐month‐old animals (IdU‐IR cells) is decreased 10‐fold compared to 3‐month‐old rats (CldU‐IR cells), the total number of CldU‐IR or IdU‐IR cells measured when animals reached senescence is similar in AU and AI and not different from untrained control animals.

These conclusions have been obtained using the hippocampal‐dependent version of the Morris water maze (measuring reference memory with variable starting points), one of the most widely used behavioral tests to study normal aging in rats; this task does not involve food restriction or the administration of shock to encourage participation of old animals and is very sensitive to the aging process (Kennard & Woodruff‐Pak, 2011; Lubec et al., 2019; Mota et al., 2019). Although inter‐individual differences in memory in aged rats have been demonstrated in other type of mazes (Barrett, Bennie, Trieu, Ping, & Tsafoulis, 2009; Temido‐Ferreira et al., 2018), in object location memory tasks (Lux, Masseck, Herlitze, & Sauvage, 2017), and in the hole‐board task (Lubec et al., 2019), we chose to use the water maze because it is the most widely used task to study the implication of adult‐born neurons in aged rats (our own work, (Bizon & Gallagher, 2003; Marrone, Ramirez‐Amaya, & Barnes, 2012)) whereas their role remains largely unexplored using the other tasks.

To map neuronal activity, Zif268 was preferred over many other markers such as c‐Fos or Arc that have been frequently used in the memory field (Gallo, Katche, Morici, Medina, & Weisstaub, 2018). Zif 268 presents the advantage to have a high “basal” expression which increases the probability to map new neurons, the number of which decreases with aging. In addition, we have previously shown using Zif 268 and c‐Fos that new neurons are recruited during both spatial memory acquisition and retrieval (Tronel, Charrier, et al., 2015; Tronel, Charrier, et al., 2015), indicating that either one or the other IEG can be used to analyze adult‐born neurons’ activation in response to learning.

To study adult‐born neuronal activity, we used immunohistochemistry, the only method available to study their role without impairing their activity. This technical approach is dependent on multiple factors that can bias sensitivity/detection of the antigens, and “old age” is a critical factor as it is accompanied with many changes—among which lipofuscin accumulation—that can interfere with antigen visualization (Moreno‐Jiménez et al., 2019) for discussion (Lucassen, Fitzsimons, Salta, & Maletic‐Savatic, 2020). Although it cannot be excluded that signal detection could be improved (Moreno‐Jiménez et al., 2019), given that all old brains within a batch were processed together from perfusion to labeling, we tend to exclude a technical bias. However, visualizing the activity of new neurons during learning using calcium imaging is now the best available technic to confirm our conclusions in behaving rats in the course of aging.

Our conclusion that the responsiveness of dentate neurons generated throughout adult life is associated with resilience to cognitive aging has been obtained using two different cohorts of rats. The first one was utilized to study Adu‐DGNs generated in senescent DG and the second one to study Adu‐DGNs generated in young adult and middle‐aged DG. When comparing the behavior of the two batches of rats, it appears that deficits in the aged‐impaired rats were much more pronounced in the first batch of animals. This cohort effect, a well‐known phenomenon in aging research (Schaie & Willis, 2015), could be related to housing conditions. Indeed, the first batch was raised in the vendor facilities until 16 months of age whereas the second one was raised in‐house. Supporting this, in our previous experiments performed in rats not aged in‐house, the difference between AU and AI was more pronounced than that observed in the second experiment (Drapeau, Montaron, Aguerre, & Abrous, 2007). However, independently of the cohort of rats the same profile of activation was observed (C = AI <AU).

While the process of neurogenesis has been well characterized in young adult rodents (Aimone et al., 2014), information about their aging and their function is less abundant (Drapeau & Abrous, 2008; Encinas & Fitzsimons, 2017; McAvoy & Sahay, 2017). The number of stem cells, their rate of proliferation, and neuronal differentiation dramatically decrease with age, and the number of immature neurons (<4 weeks old) is thus significantly decreased. Recently, the development and functional integration of these cells have been described to be delayed by age. Indeed, 3‐week‐old neurons generated in middle‐aged mice (10–14 months) displayed shorter and simpler dendrites and a dramatic reduction in spine number compared to those generated in 2‐month‐old mice and exhibited immature neuronal electrophysiological properties as revealed by the lack of functional glutamatergic synaptic inputs (Trinchero et al., 2017). In fact, their overall mature excitability and maximal glutamatergic connectivity are delayed compared to neurons born in younger animals (Trinchero, Herrero, Monzon‐Salinas, & Schinder, 2019). The long‐term destiny of adult‐born neurons generated in young adult animals has not been explored in depth. We and others have shown that contrary to what was initially hypothesized, new neurons survive for several months (Tronel, Charrier, et al., 2015) and even years in the DG (present results) and do not show signs of decline in excitability when they age: 5‐month‐old neurons are as excitable as 1‐month‐old‐cells; they can even exhibit high levels of excitability following either enriched environment exposure or induction of LTP (Ohline et al., 2018). This latest very exciting result supports our hypothesis that even when several months old, Adu‐DGNs are still plastic, they do not retire and participate in memory functions (Abrous & Wojtowicz, 2015), and even more so their persistence is not passive, but a result of their activity.

Here, we found that between middle age and senescence, the number of cells is further decreased, but then a difference between the AU and AI groups appears. Based on previous data, it is likely that the emergence of such a difference results from a difference in cell proliferation (Drapeau et al., 2003), neuronal differentiation (Drapeau et al., 2003; Qiao et al., 2019), cellular senescence (Hernandez‐Segura, Nehme, & Demaria, 2018; Micheli et al., 2019), or changes in the neurogenic niche and/or to the systemic milieu (Mahmoudi, Xu, & Brunet, 2019; Villeda et al., 2011). Interestingly, we have shown that the senescent neurogenic niche is capable to rejuvenate upon removal of corticosterone (Montaron et al., 1999) or addition of pregnenolone sulfate (Mayo et al., 2005), indicating that neural stem cells are not depleted and keep their abilities to divide.

The main finding of our study is that there is a strong link between the ability for newborn cells to be recruited by learning and memory abilities in aged rats. Indeed, the percentage of adult‐born cells expressing Zif268 was higher in animals that learned the task compared to animals that did it to a lesser extent. This finding is in accordance with our previous data showing that (a) when compared to control rats (naïve rats or rats trained to find a visible platform), adults required to use an hippocampal‐dependent strategy in the water maze (or the dry maze) exhibit an increased percentage of mature adult‐born neurons expressing Zif268 (Tronel, Charrier, et al., 2015), and (b) ablating mature adult‐born neurons generated 4 months before training (when animals were 3 months old) delays the ability of rats to learn such a task (Lemaire et al., 2012). In the present experiment, the percentage of adult‐born cells expressing Zif268 in each experimental group was similar in the three neuronal populations studied. It was thus independent of the age of the animals at the time of labeling (3, 12, and 18 months) and of the age of the cells at the time of training (4, 10, and 19 months). It was also independent of the total number of XdU cells. Note that even if in the first batch of animals, fifty percent of BrdU‐IR cells differentiated into neurons, 96% of Zif268 cells were neurons suggesting that all BrdU‐Zif268 cells were meant to be activated new neurons.

It could be argued that neurons born during development, which represent a major part of the DG, are also involved in differences in spatial memory abilities in old age. However, three arguments seem to rule out this hypothesis. First, the total number of granule cells is similar in AU and AI groups (Drapeau et al., 2003, 2007; Rapp & Gallagher, 1996). Second, if neurons generated during development (prenatal and postnatal periods) were activated by spatial learning, given their high numbers, differences in the total number of Zif268 cells should have emerged as a function of the cognitive status. Third, we have shown that neurons born in neonates are not activated by spatial learning when they are mature compared to neurons of the same age born in adults. Indeed, the former are not recruited by spatial learning in the water maze when animals are tested at 7 months (Tronel, Charrier, et al., 2015). Here, we extended this observation showing that neurons born during the juvenile (PN28) or the embryonic (E18.5/19.5) period are not differentially recruited in good and bad learners. Recently, we began to explore the reasons for which developmentally and adult‐generated neurons do not respond in the same way. By comparing the dendritic arbor of neurons born at different ontogenetic stages (embryonic, neonatal, adolescence, adulthood), we found that they display distinct morphological features and also different location (Kerloch, Clavreul, Goron, Abrous, & Pacary, 2019; Mathews et al., 2010) that may underlie different inputs and functions.

One question that we did not address is whether the three neuronal populations studied participate to the same extent to learning. To address this point, sophisticated models that allow to selectively tag new neurons generated within a defined period of time (adulthood, middle age, or senescence) and to ablate them during training performed at senescence are required. One possibility would be to take advantage of the recently developed pharmacogenetic or optogenetic approaches or in order to tag specifically neurons born in young adult rats and manipulate them when animals have reached senescence.

A previous study has shown that 4‐month‐old neurons generated in old rats exhibiting spatial memory deficits are recruited in response to spatial exploration behavior with the same probability than 4‐month‐old neurons generated in aged good learners or in young adult rats (Marrone et al., 2012). From this dataset, it was concluded that disrupted information processing at old age may be linked to a reduced number of adult‐generated granule neurons and not to a deficit in their functionality. However, in this study the activation of adult‐generated neurons was evaluated in response to a simple form a learning (spatial exploration). Taking the present data into consideration, we rather suggest that adult‐born neurons in AU are sufficiently connected to integrate simple stimulations generated during simple forms of learning but insufficiently integrated to process the complex stimulations generated during spatial navigation.

Zif268 is known to be regulated in an activity‐dependent manner by learning (for review, see Gallo et al., 2018; Veyrac, Besnard, Caboche, Davis, & Laroche, 2014). It is overexpressed in response to different types of learning in distinct structures and circuits that are processing the ongoing information, and several arguments indicate that it is required for the stabilization (and not acquisition) of long‐lasting memories. Although the mechanisms are not fully understood, the activation of Zif268 may strengthen/stabilize the memory trace. It can be hypothesized that during learning the activation of Zif268 in adult‐born neurons may be involved in the formation, stabilization, and reactivation of place cells in the hippocampal network, events known to support spatial learning.

Here, we hypothesize that adult‐born neurons that do not exhibited activity‐dependent regulation of Zif268 become functionally silent in the course of aging, leading to memory deficits. Although the firing patterns that are sufficient to induce Zif268 in adult‐born neurons in “behaving” animals are so far unknown, adult‐born neurons silencing may have several origins. It may result from a loss of synaptic inputs (Geinisman, Toledo‐Morrell, & Morrell, 1986) altering the ability to fire properly (Ahlenius, Visan, Kokaia, Lindvall, & Kokaia, 2009); these synaptic alterations of Adu‐DGNs could be linked to the acceleration of senescence through epigenetic changes (Penner, Roth, Barnes, & Sweatt, 2010), decreased autophagy activity (Glatigny et al., 2019), or changes of the local and systemic milieu (Villeda et al., 2011).

The HPA axis (and corticosterone) deserves a special attention in relation to its role in age‐related memory disorders (Sapolsky, 1992). In his pioneer work, Issa and colleagues showed that AU rats were characterized by high level of corticosterone in basal condition and in response to a restraint stress (Issa, Rowe, Gauthier, & Meaney, 1990). We replicated and extended this finding by showing that animals with the heaviest adrenal glands, indicative of chronic HPA axis hyperactivity, exhibited the worst memory performance and the lowest number of proliferating cells or 3‐week‐old surviving cells in comparison with AU rats (Drapeau et al., 2003). Although some reports failed to support a relationship between cell birth and corticosterone levels during aging (using different rat strain (Heine, Maslam, Joels, & Lucassen, 2004), we can hypothesize that in the present experiment AU and AI rats exhibit different HPA axis activity both in basal condition and in response to training. This raises the possibility that differences in the reactivity of adult‐born neurons in these animals are related to differences in the activity of the HPA axis. The influence of learning in the water maze on HPA axis activity is largely unknown at the exception of one study showing that corticosterone secretion is increased (in adult rats) immediately after the first training session and that corticosterone has pro‐mnesic effect on learning (Sandi, Loscertales, & Guaza, 1997). Whether or not learning still increases corticosterone at the end of training when animals get habituated to the task is not known. But independently of this, AI exhibit a hyperactive HPA axis and it cannot be excluded that excessive levels of corticosterone may impair adult‐born neurons’ functioning and memory processing. As a consequence, blocking/removing glucocorticoid receptors expressed by adult‐born neurons generated early in life at the time of training may increase their responsiveness to learning and promote memory. In this context, we have previously shown that blocking age‐related increased in HPA axis activity form middle‐age onward rejuvenates memory and the neurogenic niche in senescent subjects (Montaron et al., 2006).

Other strategies are also promising to prevent the development of memory disorders during the course of aging. To name a few, transfusion of aging individuals with young plasma, using metabolic drugs (rapamycin/metformin) or intracellular metabolic cascade (mTor), increasing mitochondrial fitness, ablating senescent cells (with senolytics), cellular reprogramming through epigenomic remodeling, transplanting cells, or other noninvasive environmental approaches may promote successful brain aging (Mahmoudi et al., 2019; Villeda et al., 2011) (Fakouri, Hansen, Desler, Anugula, & Rasmussen, 2019; Munoz‐Espin & Serrano, 2014; Shetty, Kodali, Upadhya, & Madhu, 2018).

In conclusion, our results highlight the importance of neurons born throughout adult life in providing resilience to age‐related memory disorders. They reveal a novel perspective for developing therapies to promote resilience to age‐related memory disorders or to rejuvenate the DG by acting throughout adult life on adult‐born dentate neurons.

## 4 EXPERIMENTAL PROCEDURES

### 4.1 Animal

For these experiments, a total of 96 male Sprague Dawley rats (OFA, Janvier, France) were used. Animals were housed collectively until behavioral testing under a 12‐hr:12‐hr light/dark cycle with ad libitum access to food and water. Temperature (22°C) and humidity (60%) were kept constant.

*In the first experiment*, male rats (*n* = 19) were 16 months old on delivery. *In the second experiment*, rats (*n* = 32) were 2 months old on delivery. *In the third and fourth experiments*, rats (*n* = 25) were 21 days old on delivery. In the fifth experiment, pregnant Sprague Dawley female rats (*n* = 4) were individually housed in transparent cages. After delivery, litters were raised by their biological mothers until weaning (21 days after birth). After weaning, only the male progeny (*n* = 20) was kept. Rats were individually housed before the beginning of behavioral training. Animals with a bad general health status or tumors were excluded. Experimental procedures have been planned respecting the European directive of the parliament and the conceal of September 22, 2010 (2010/63/UE, 5012006A).

### 4.2 Thymidine analog injections

Newly born cells were labeled by the incorporation of synthetic thymidine analogs (XdU, Sigma‐Aldrich, Table 1). *In the first experiment*, rats were injected with 5‐bromo‐2′‐deoxyuridine (BrdU) according to a previously described protocol (Drapeau et al., 2003, 2007). These animals received one daily BrdU injection (50 mg kg^‒1^ day^‒1^; ip) for 5 days when 18 months old, that is, 4 months before training. *In the second experiment*, rats received five injections of 5‐chloro‐2′‐deoxyuridine (CldU) when 3 months old and five injections of 5‐iodo‐2′‐deoxyuridine (IdU) when 12 months old (Dupret et al., 2007), both at equimolar doses of 50 mg BrdU/kg. *In the third and fourth experiments,* animals received one injection of CldU when 28 days old (equimolar dose of 50 mg BrdU/kg). *In the fifth experiment,* pregnant female rats received two injections of 5‐chloro‐2'‐deoxyuridine (CldU, equimolar dose of 50 mg BrdU/kg 50 mg/kg) at E18.5 and E19.5.

### 4.3 Water‐maze training

Rats were tested in the water maze when 22 months old (experiments 1, 2, and 4) or 15 months old (experiments 3 and 5). The apparatus consisted of a circular plastic swimming pool (180 cm diameter, 60 cm height) that was filled with water (20 ± 1°C) rendered opaque by the addition of a white cosmetic adjuvant. Before the start of training, animals were habituated to the pool for 2 days for 1 min per day. During training, the *Learning* group (L) was composed of animals that were required to locate the submerged platform, which was hidden 1.5 cm under the surface of the water in a fixed location, using spatial cues available within the room. Rats were all trained for four trials per day (90 s with an inter‐trial interval of 30 s and released from 3 different starting points that varied randomly each day). If an animal failed to locate the platform, it was placed on it at the end of the trial. The time necessary to reach the platform was recorded using a video camera that was secured to the ceiling of the room and connected to a computerized tracking system (Videotrack, Viewpoint). Daily results were analyzed in order to rank animals according to their behavioral score calculated over the last 3 days of training (when performances reached an asymptotic level). The behavioral scores calculated over the whole training duration of aged‐unimpaired (AU) rats were below the median of the group, whereas those of aged‐impaired (AI) animals were above the median of the group. Control groups consisted of animals that were transferred to the testing room at the same time and with the same procedures as trained animals but that were not exposed to the water maze.

### 4.4 Immunohistochemistry

Animals were sacrificed 90 min after the last trial (Table 1). The different age‐matched control groups were sacrificed within the same period. Free‐floating sections (50 µm) were processed using a standard immunohistochemical procedure to visualize thymidine analogs (BrdU, CldU, IdU) on alternate one‐in‐ten sections using different anti‐BrdU antibodies from different vendors (for BrdU: 1/200, Dako; CldU: 1/500, Accurate Chemical; IdU: 1/200, BD Biosciences) and Zif268 (1:500, Santa Cruz Biotechnology). The number of XdU‐immunoreactive (IR) cells in the granule and subgranular layers (GCL) of the DG was estimated on a systematic random sampling of every tenth section along the septo‐temporal axis of the hippocampal formation using a modified version of the optical fractionator method. Indeed, all XdU‐IR cells were counted on each section and the resulting numbers were tallied and multiplied by the inverse of the section sampling fraction (1/ssf = 10 for BrdU and IdU cells that were counted in both sides of the DG and 1/ssf = 20 for CldU‐IR cells that were counted in the left side). The number of Zif268‐IR cells (left side) was determined using a 100× lens, and a 60 × 60 µm frame at evenly spaced x‐y intervals of 350 µm by 350 µm with a Stereo Investigator software (Microbrightfield).

### 4.5 Activation of new cells

The activation of adult‐born cells was examined using immunohistofluorescence. To visualize cells that incorporated thymidine analogs, one‐in‐ten sections were incubated with different anti‐BrdU antibodies (BrdU and CldU, rat primary antibodies at 1/200 Accurate Chemical; IdU, mouse primary antibodies at 1/200, BD Biosciences). Sections were also incubated with Zif268 (rabbit, 1:500, Santa Cruz Biotechnology). Bound antibodies were visualized, respectively, with Cy3‐goat anti‐rat (1:1,000, Jackson) or Cy3‐goat anti‐mouse (1:1,000, Jackson) and Alexa 488 goat anti‐rabbit antibodies (1:1,000, Jackson). CldU‐Zif268 and IdU‐Zif268 labeling were analyzed on different sections because of some cross‐reactivity between secondary antibodies made in mice or rat (Figure 1 in Tronel, Charrier, et al., 2015). All BrdU^‒^, CldU^‒^, or IdU‐labeled cells expressing Zif268 (one side) were analyzed using a confocal microscope with HeNe and Arg lasers (Leica, DMR TCSSP2AOBS), with a plane apochromatic 63X oil lens (numerical aperture 1.4; Leica). The percentage of BrdU^‒^, CldU^‒^, or IdU‐labeled cells that expressed Zif268 was calculated as follow: (Nb of Xd ^+^/IEG^+^ cells)/[(Nb of XdU^+^/IEG^‐^ cells) + (Nb of XdU^+^/IEG^+^ cells)] × 100. All sections were optically sliced in the Z plane using 1‐µm interval, and cells were rotated in orthogonal planes to verify double labeling.

### 4.6 Analysis of phenotype

One‐of‐ten series was incubated with a rat monoclonal anti‐BrdU antibody (1/200, Accurate Chemical) and with a mouse monoclonal anti‐NeuN antibody (1:500, Millipore). Bound anti‐BrdU and anti‐NeuN antibodies were visualized with a Cy3‐goat anti‐rat (1:1,000, Jackson) and an Alexa 488 goat anti‐mouse IgG antibody (1:1,000, Jackson). The phenotype of IdU‐IR cells and CldU‐IR cells was determined using rabbit anti‐calbindin antibodies (1/200, Millipore) that were revealed with Alexa 488 goat anti‐rabbit IgG antibodies (1/500, Jackson). We also analyzed the phenotype of Zif268 cells by incubating one‐in‐ten sections with a rabbit anti‐Zif268 antibody (1:500, Santa Cruz Biotechnology) and a mouse monoclonal anti‐NeuN antibody (1:500, Millipore). Bound anti‐Zif268 and anti‐NeuN antibodies were visualized with a Cy3‐goat anti‐rabbit (1:1,000, Jackson) and an Alexa 488 goat anti‐mouse IgG antibody (1:1,000, Jackson).

### 4.7 Statistical analysis

All data are expressed as mean ± *SEM*. Data were analyzed using an ANOVA or Student's *t* test (2 tails) when necessary.

## CONFLICT OF INTEREST

The authors declare no conflict of interest.

## AUTHOR CONTRIBUTIONS

MFM performed experiments. VC analyzed the recruitment of DGNs of rats from batches 4 and 5 and revised the paper. NB analyzed the recruitment of DGNs of animals from batch 3. PG performed experiment from batch 1. DNA conceived experiments, analyzed data and wrote the paper.

## Supporting information

Fig S1

Table S1

## Data Availability

The authors elect to not share data.
