# Supplementary figures and images for: Responsiveness of dentate neurons generated throughout adult life is associated with resilience to cognitive aging
Source: Aging Cell. 2020 Jun 29;19(8):e13161. doi: 10.1111/acel.13161 (PMC7431828; doi:10.1111/acel.13161)

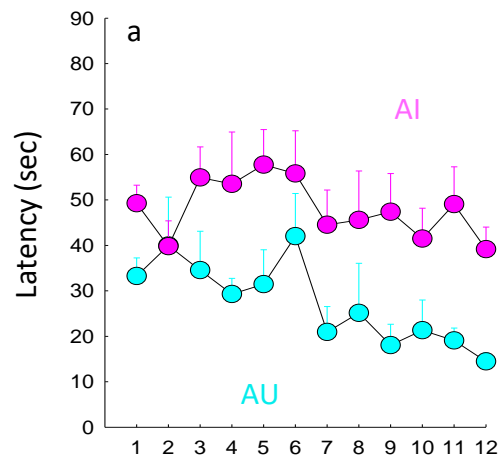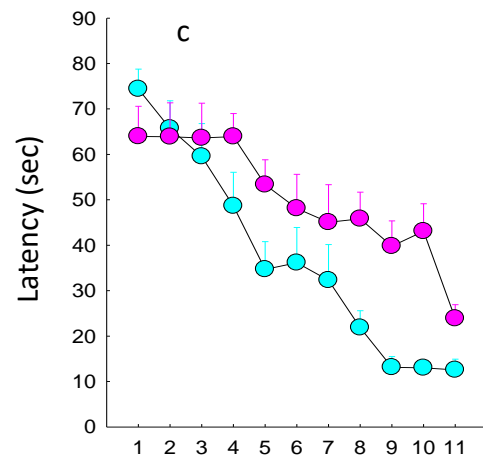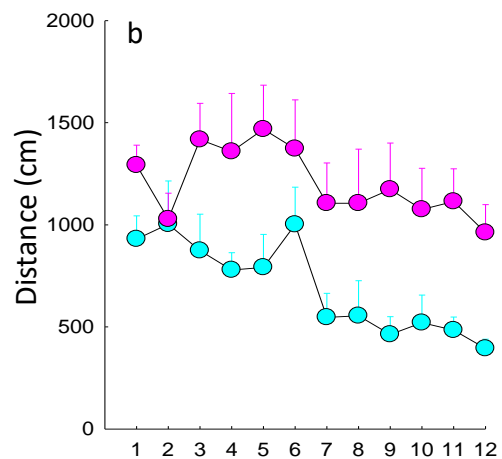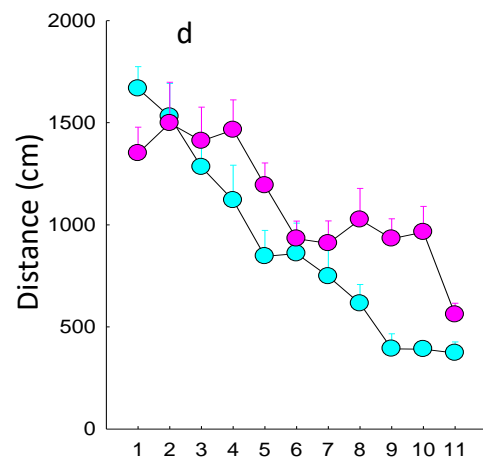

Supplement: Supplementary file 1 — Fig S1 [file ACEL-19-e13161-s001.pdf]
