# Supplementary material for: Responsiveness of dentate neurons generated throughout adult life is associated with resilience to cognitive aging
Source: Aging Cell. 2020 Jun 29;19(8):e13161. doi: 10.1111/acel.13161 (PMC7431828; doi:10.1111/acel.13161)
Supplement: Supplementary file 2 — Table S1 [file ACEL-19-e13161-s002.docx]

| **Experiments** | **Batch** | **Nb of Cldu-IR cells** | **Statistical analysis** | **% of CldU-Zif-268IR cells** | **Statistical analysis** |
| --- | --- | --- | --- | --- | --- |
| **Ado**-DGNs | 3 | AU : 10496 ±1219  AI : 8626 ± 1027 | t_8_=1.17, p=0.27 | AU : 2.08 ± 0.63  AI : 3.21 ± 0.76 | t_8_=-1.14, p=0.29 |
| **Ado**-DGNs | 4 | AU : 6928 ± 2131  AI : 7724 ± 1473 | t_8_=-0.31, p=0.77 | AU : 3.52 ± 1.51  AI : 2.62 ± 0.13 | t_8_=0.59, p=0.57 |
| **Embryo**-DGNs | 5 | AU : 160510 ± 10375  AI : 149371 ± 25974 | t_11_=0.42, p=0.68 | AU : 7.84 ± 0.63  AI : 6.74 ± 0.35 | t_11_=1.45, p=0.17 |

**Table 1 :** DGNs produced in adolescent rats or embryos are not activated by spatial learning in aged rats.
